# Antibiotics reduce bacterial load in *Exaiptasia diaphana*, but biofilms hinder its development as a gnotobiotic coral model

**DOI:** 10.1099/acmi.0.000314

**Published:** 2022-01-24

**Authors:** Leon M. Hartman, Linda L. Blackall, Madeleine J. H. van Oppen

**Affiliations:** ^1^​ School of BioSciences, The University of Melbourne, Melbourne, VIC, Australia; ^2^​ Swinburne University of Technology, Hawthorn, VIC, Australia; ^3^​ Monash University, Clayton, VIC, Australia; ^4^​ Australian Institute of Marine Science, Townsville, QLD, Australia

**Keywords:** bacterial load, coral, ddPCR, *Exaiptasia*, gnotobiotic

## Abstract

Coral reefs are declining due to anthropogenic disturbances, including climate change. Therefore, improving our understanding of coral ecosystems is vital, and the influence of bacteria on coral health has attracted particular interest. However, a gnotobiotic coral model that could enhance studies of coral–bacteria interactions is absent. To address this gap, we tested the ability of treatment with seven antibiotics for 3 weeks to deplete bacteria in *Exaiptasia diaphana*, a sea anemone widely used as a coral model. Digital droplet PCR (ddPCR) targeting anemone *Ef1-α* and bacterial 16S rRNA genes was used to quantify bacterial load, which was found to decrease six-fold. However, metabarcoding of bacterial 16S rRNA genes showed that alpha and beta diversity of the anemone-associated bacterial communities increased significantly. Therefore, gnotobiotic *E. diaphana* with simplified, uniform bacterial communities were not generated, with biofilm formation in the culture vessels most likely impeding efforts to eliminate bacteria. Despite this outcome, our work will inform future efforts to create a much needed gnotobiotic coral model.

## Introduction

The sea anemone *Exaiptasia diaphana* (previously *Aiptasia pallida* [1, 2]) has become an important coral model as its intracellular symbiosis with photosynthetic algae of the family Symbiodiniaceae makes it useful for studying host–symbiont relationships [3, 4]. The breakdown of this relationship (i.e. bleaching) has been particularly well studied [5–9] due to an increase in the frequency of mass coral bleaching events linked to climate change [10].

*E. diaphana*’s ability to survive in a Symbiodiniaceae-free (i.e. aposymbiotic) state has clarified metabolic processes within cnidarians by separating the host and its algal symbiont to reveal the role of each in nutrient transfer [11–13] and their response to environmental stress [14–16]. However, studies that have investigated the relationship between *E. diaphana* and Symbiodiniaceae have often ignored the influence of bacteria on the holobiont, a functional entity comprising the host and all its microbial partners [17].

The bacterial component of the holobiont influences host health, for example through its involvement in nutrient cycling [18–21] and pathogen protection [22–24]. Therefore, removing bacteria from the holobiont represents an important next step in the elucidation of host and symbiont interdependence [25]. Although germ-free Symbiodiniaceae cells have been created [26], similar coral cultures or cell lines have not [25]. *E. diaphana* may be able to fill this gap.

There has been one report of germ-free *E. diaphana*, wherein anemones were exposed to two antibiotics to render them ‘aseptic’ [27]. Germ-free status was determined by culture methods and microscopy. However, as many bacteria cannot be cultured [28], and the extent to which an anemone can be screened by microscopy is limited, the germ-free status of the anemones is uncertain. In fact, creating germ-free *E. diaphana* might not be feasible as the anemones could require bacteria for normal host health and development, as in many other organisms [29–31]. Thus, *E. diaphana* that harbour reduced bacterial communities could represent a practical alternative to germ-free cultures. Strictly, these anemones would be described as gnotobiotic, that is, organisms with depleted microbial communities that are simple (i.e. possessing low individual, or alpha, diversity), uniform (i.e. possessing low inter-individual, or beta, diversity) and precisely defined [32].

In a recent step towards development of gnotobiotic *E. diaphana*, a method for bacterial depletion was reported [33, 34]. Depletion was achieved by exposure to four antibiotics with different mechanisms of action, and detection of bacteria in the treated anemones was by culture methods and PCR. However, depletion was only maintained with continuous treatment. In addition, bacterial load was not quantified, and the bacterial communities were not characterized, leaving the extent of the depletion and the uniformity and composition of the resulting bacterial communities unknown. Consequently, the efficacy of antibiotic approaches for generating gnotobiotic *E. diaphana* cultures remains unclear.

Here, we describe our efforts to produce gnotobiotic *E. diaphana* by exposure to antibiotics. Our aim was to determine whether anemone bacterial load could be reduced and the bacterial communities made uniform by antibiotic treatment. Further, by presenting our methods, we hope to assist other researchers seeking to create gnotobiotic *E. diaphana*, a resource that could help clarify the relationship between cnidarians and their bacterial associates.

## Methods

### Experimental set-up and antibiotic treatment

Clonal adult *E. diaphana* anemones (*n*=72; genotype AIMS2) were haphazardly selected from a single tank in the University of Melbourne (UoM) culture collection [35]. Individuals were transferred into single wells within sterile 12-well plates (CLS3513; Corning) where they were maintained in seawater reconstituted from Red Sea Salt (R11065) with reverse osmosis water at a salinity of ~34 p.p.t. The lidded plates were kept in clear, sterile plastic zip-lock bags to prevent contamination and were randomly positioned in a Hi-Point 740 incubator (Thermo Fisher) at 26 °C with lighting at ~33 µmol m^–2^ s^–1^ on a 12 h:12 h light–dark cycle. During a 1 week acclimation period the *E. diaphana* were fed twice with freshly hatched *Artemia salina* nauplii (Salt Creek, Premium GSL), and the water was changed three times (Table 1). After the acclimation period, antibiotic treatment and sampling commenced. All subsequent operations were performed using aseptic techniques. During the treatment period, water changes were performed with Red Sea Salt water sterilized by autoclaving (hereafter, ‘sRSS-water’). Treatment was scheduled to coincide with water changes to ensure the anemones were constantly exposed to antibiotics. Sampling was performed more frequently in the first week of treatment to track the impact of the antibiotics on the intact bacterial communities. When sampling coincided with treatment or water changes, samples were collected first.

**Table 1.** *E. diaphana* maintenance, antibiotic treatment and sampling schedule

Monday	Tuesday	Wednesday	Thursday	Friday
Transfer *E. diaphana* Hatch *A. salina*	Feed *E. diaphana*	Change water	Hatch *A. salina*	Change water Feed *E. diaphana*
**Day 0** **Sampling** Change water **Treatment** Hatch *A. salina*	**Day 1** **Sampling** Feed *E. diaphana*	**Day 2** Change water **Treatment**	**Day 3** **Sampling** Hatch *A. salina*	**Day 4** Change water **Treatment** Feed *E. diaphana*
**Day 7** **Sampling** Change water **Treatment** Hatch *A. salina*	**Day 8** Feed *E. diaphana*	**Day 9** Change water **Treatment**	**Day 10** Hatch *A. salina*	**Day 11** Change water **Treatment** Feed *E. diaphana*
**Day 14** **Sampling** Change water **Treatment** Hatch *A. salina*	**Day 15** Feed *E. diaphana*	**Day 16** Change water **Treatment**	**Day 17** Hatch *A. salina*	**Day 18** Change water **Treatment** Feed *E. diaphana*
**Day 21** **Sampling**				

On treatment days, half the *E. diaphana* were exposed to antibiotics selected for their different mechanisms of action and activity against Gram-positive or Gram-negative bacteria, and their previous use on cnidarians, Symbiodiniaceae, sponges and *A. salina* (Table 2). Maximum tolerable concentrations were determined in pre-treatment testing by exposing *E. diaphana* to increasing dilutions of the combined antibiotics until they maintained normal appearance, growth and feeding over an 18 day test period.

**Table 2.** Antibiotics used to deplete bacteria in *E. diaphana* and *A. salina*

Antibiotic	Concentration (µg ml^–1^)	Target/mechanism of action	Gram +/− activity	References to prior use
Carbenicillin	25	dd-Transpeptidase/inhibits cell wall synthesis	−	[33, 97–100]*
Chloramphenicol	25	23S rRNA/inhibits protein synthesis	+/−	[33, 97, 100, 101]
Nalidixic acid	15	Gyrase/inhibits DNA replication	−	[33, 102, 103]
Neomycin	10	30S rRNA assembly/inhibits protein synthesis	+/−	[100, 104, 105]
Polymyxin B	10	Cell wall/increases cell wall permeability	−	[100, 104, 105]
Rifampicin	10	RNA polymerase/inhibits transcription	+	[33, 101, 105]
Streptomycin	25	16S rRNA/inhibits protein synthesis	+	[26, 27, 97, 99, 101, 104–106]

*References to the use of Penicillin family antibiotics with the same mechanism of action.

Control *E. diaphana* were fed with *A. salina* hatched in sRSS-water. Treated *E. diaphana* were fed with *A. salina* hatched in sRSS-water containing antibiotics at concentrations matching those used for *E. diaphana*.

### Sampling and DNA extraction

On sampling days, six control and six treated *E. diaphana* were killed to measure changes in bacterial load and community composition. When collected, each anemone was gently passed two or three times through the tip of a sterile transfer pipette to remove loosely attached debris. Three 50 µl aliquots of a dense suspension of control and treated *A. salina* nauplii were also collected for bacterial load and community analyses. All samples were snap frozen and stored at –80 °C until processing. DNA was extracted from the bacterial analysis samples using a salting out protocol [36] modified according to Hartman *et al.* [37].

### Bacterial load assessment (B/H ratio)

Bacterial load in *E. diaphana* and *A. salina* was quantified according to the number of bacterial gene copies to host gene copies in each DNA extract. The copy number data were obtained by digital droplet PCR (ddPCR) to allow calculation of the bacteria/host (B/H) cell ratio [38] for each sample. This approach has also been used to analyse bacterial load in insect samples with low mass and volume [39–41], with the use of a ratio accounting for differences in sample size. Primers targeting single-copy reference genes in *E. diaphana* and *A. salina* were used for host cell quantification (Table 3). The translation elongation factor 1 alpha gene (*Ef1-α*) was used for *E. diaphana*, and the beta actin gene (*ß-actin*) was used for *A. salina*. Primers targeting a conserved 98-nt sequence between the V2 and V3 regions of the bacterial 16S rRNA gene were used to estimate bacterial cell numbers as they produced small amplicons and no non-specific PCR product, which was essential for optimal ddPCR performance. No correction was made for 16S rRNA gene copy number, and hence the method is semi-quantitative.

**Table 3.** Primers used in the present study to estimate host and bacterial cell numbers

Target (gene)	Primer name	Primer sequence	Annealing temperature (°C)	Product size (nt)	References
*E. diaphana* (*Ef1-α*)	Ef1-α-fwd	AGCACTGAGCCACCATACAG	60	88	[107]
	Ef1-α -rev	TTGGGTTATAGCCGGTCTTC	60		[107]
*A. salina* (*ß-actin*)	art-actin-fwd	GGTCGTGACTTGACGGACTATCT	60	147	[108–111]
	art-actin-rev	AGCGGTTGCCATTTCTTGTT	60		[108–111]
Universal bacteria (conserved inter V2–V3 16S rRNA gene region)	259-fwd	GGTAAHRGCYYACCAAG	54	98	[112]
	357-rev	CTGCTGCCTCCCGTAGGAG	54		Reverse complement of ‘primer 1’ [113]

Before performing ddPCR, DNA was restriction enzyme-digested to improve droplet encapsulation of DNA fragments and signal generation from low-concentration bacterial DNA [42]. Sample DNA was digested for 1.5 h at 37 °C in a volume of 20 µl comprising 7 µl sterile water, 2 µl of 10× restriction enzyme buffer, 10 µl DNA extract, and 1 µl (~20 U) *Hin*dIII (R3104S-HF; New England BioLabs). The digested DNA was then quantified by PicoGreen (P11496; Thermo Fisher) and diluted ≥1:4 to 10–20 ng µl^–1^ to create practical working concentrations and prevent PCR inhibition by the enzyme buffer.

ddPCRs for each DNA sample were prepared in an initial volume of 44 µl comprising 24 µl EvaGreen Supermix (QX200; Bio-Rad), sterile water and ~30 ng of digested DNA to ensure that DNA concentrations for each bacteria–host reaction pair were within the dynamic range of the ddPCR system. The mixture was then split into two 22 µl aliquots, one for host cell quantification (i.e. *E. diaphana* or *A. salina*) and one for bacteria. One microlitre each of the appropriate 5 µM forward and reverse primers (Table 3) was then added to each reaction aliquot, giving final primer concentrations of ~200 nM and volumes of 24 µl. From each 24 µl volume, 20 µl was loaded into a DG8 cartridge (1864008; Bio-Rad), followed by 70 µl of droplet generation oil for EvaGreen (1864005; Bio-Rad), and droplets were generated in a droplet-generator (QX200; Bio-Rad). A volume of 40 µl of generated droplets per reaction was then transferred to a 96-well plate and foil-sealed (1814040; Bio-Rad) with a thermal plate-sealer (PX1; Bio-Rad). One no-template control (NTC) reaction was included per plate. Thermal cycler settings were optimized according to Witte *et al.* [43]: one cycle at 95.0 °C for 5 min; 50 cycles at 95 °C for 1 min + 54 °C or 60 °C (see Table 3) for 2 min; one cycle at 4.0 °C for 5 min; one cycle at 90 °C for 5 min; 12 °C hold. All ramp rates were 1 °C s^–1^. Droplets were read on a Bio-Rad QX200 droplet reader, and fluorescence data were analysed in QuantaSoft v1.7.4.0917 (Tables S1 and S2, Fig. S1, available in the online version of this article).

### Sample and data processing for metabarcoding analysis

In preparation for bacterial community analysis by metabarcoding, sample DNA was amplified by end-point PCR using primers with Illumina overhang sequences (underlined) targeting the V5–V6 regions of the 16S rRNA gene: 784F 5′-TCGTCGGCAGCGTCAGATGTGTATAAGAGACAGAGGATTAGATACCCTGGTA-3′; 1061R 5′-GTCTCGTGGGCTCGGAGATGTGTATAAGAGACAGCRRCACGAGCTGACGAC-3′ [44]. Triplicate PCRs were performed in 20 µl volumes comprising 1 µl DNA extract, 10 µl MyTaq HS Mix polymerase (Bioline), 0.5 µl of 10 µM 784F, 0.5 µl of 10 µM 1061R, and 8 µl MilliQ water. Thermal-cycler settings were: one cycle at 95.0 °C for 3 min; 30 cycles at 95.0, 55.0 and 72.0 °C for 15 s each; one cycle at 72 °C for 3 min; 12 °C hold. Each triplicate was pooled, then checked by 1% agarose gel electrophoresis. No-sample DNA extractions and no-template PCRs were performed to identify contaminants introduced during sample preparation. A volume of 25 µl of pooled PCR product from each sample was sent to the Ramaciotti Centre for Genomics (RCG), Sydney, Australia, for sequencing on a single Illumina MiSeq 2×250 bp run. RCG performed PCR product clean-up and normalization as part of library preparation prior to sequencing. The resulting Illumina MiSeq data were deposited in the NCBI Sequence Read Archive under BioProject accession number PRJNA698456. Demultiplexed MiSeq reads were joined in QIIME2 v2020.8.0 [45]. Denoising, chimera filtering and trimming was performed using DADA2 within the QIIME2 environment [46]. Resulting amplicon sequence variants (ASVs) >289 nt were removed. Taxonomy was assigned to the remaining ASVs against the silva database (v132) trained with a naïve Bayes classifier [47–50]. Unidentified ASVs, and ASVs identified as mitochondrial or chloroplast sequences were removed.

### Data analyses

Data analyses were performed in R v4.0.3 [51], with differences considered significant at α=0.05 unless otherwise stated. The ddPCR count data were imported into R and the B/H ratios were calculated and plotted over time with the R package ggplot2 [52]. Overall differences in B/H were assessed by generalized least square (GLS) models with the R package nlme [53]. If the B/H data met homogeneity of variance [54] and normality criteria [55], Student’s *t*-test [56] was used to assess differences between samples, otherwise the Mann–Whitney *U* test [57] was used. Tabulated ASV counts, taxonomic and meta data were imported and converted into a phyloseq object for bacterial community analyses [58]. Rarefaction curves were generated with the R package vegan [59] to assess whether the metabarcoding samples had been sequenced sufficiently to capture species diversity. Putative contaminating ASVs were identified with the R package decontam [60] and removed. The ASV counts for each *E. diaphana* sample were multiplied by the corresponding B/H ratio ×10^3^ to convert counts to 16S rRNA gene copies per host cell ×10^3^ (hereafter, 16S/H × 10^3^), thus producing absolute abundance values corrected for *E. diaphana* size differences [61, 62]. To identify samples with highly divergent bacterial compositions, the *E. diaphana* bacterial community data were visualized in a non-metric multidimensional scaling (nMDS) ordination based on Bray–Curtis dissimilarity [63], and a principal component analysis (PCA) ordination of centre log-ratio (CLR)-transformed data using vegan [59] and the R package mixOmics [64], respectively. Alpha diversity metrics for the *E. diaphana* bacterial communities were calculated in vegan [59] and plotted over time with ggplot2 after sub-sampling the data to 48 176 B/H-converted ASV counts per sample. Bacterial community richness was described by number of observed ASVs per *E. diaphana* sample. Community evenness was described using Simpson’s index [65]. General alpha diversity was described using Shannon’s index [66]. Overall differences in the alpha diversity metrics were assessed by GLS models and sample-wise differences were assessed using Mann–Whitney *U* tests, as above. Relationships between the untreated and treated *E. diaphana* bacterial communities at Day 0 and Day 21 were visualized in nMDS and PCA ordinations, as above, and differences between them were tested using generalized linear models (GLMs) in the R package mvabund [67]. Common and unique ASVs in the Day 21 control and treated *E. diaphana* were visualized in petal diagrams to compare the complexity of their bacterial communities. ASVs with an absolute abundance ≥1000 16S/H × 10^3^ in the treated *E. diaphana* at all timepoints were identified to highlight antibiotic-tolerant bacteria [68]. The tolerant ASV abundances were plotted with ggplot2. To assess their possible origin or location, they were then compared to the *A. salina* bacterial community data from Maire *et al.* [69], which identified the bacteria associated with Symbiodiniaceae isolated from *E. diaphana* in the UoM culture collection.

## Results

### Changes in bacterial load (B/H ratio)

**Fig. 1.**
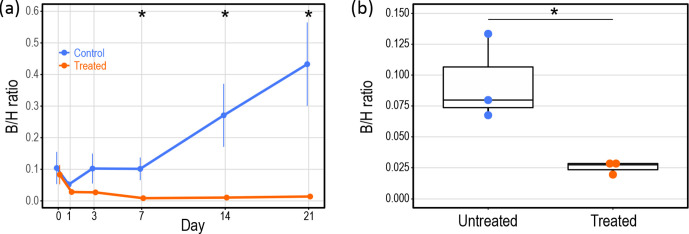
Effect of antibiotic treatment on the bacterial load (B/H) of *E. diaphana*, and the *A. salina* feed stock. (**a**) Temporal change in B/H in *E. diaphana*. For each datapoint, *n*=5–6. Error bars±1 sem. (**b**) B/H in the untreated and treated *A. salina* feed stock, *n*=3. Asterisks indicate significant difference, α=0.05.

All antibiotic-treated *E. diaphana* survived until being killed and were phenotypically comparable to the control *E. diaphana* with respect to tentacle extension, mobility and feeding behaviour throughout the experiment. However, significant changes in B/H occurred in the *E. diaphana* according to treatment (GLS, *χ* ^2^=22.64, *P*<0.001) and time (GLS, *χ* ^2^=14.52, *P*<0.001) (Fig. 1a). The antibiotic-treated *E. diaphana* underwent a three-fold decrease in B/H from Day 0 to Day 1, and a significant six-fold decrease overall (Mann–Whitney, *P*=0.030). Despite the decrease, B/H in the treated *E. diaphana* remained above zero through to Day 21 indicating that bacteria were not completely eliminated. The B/H of the control *E. diaphana* also decreased from Day 0 to Day 1, but then recovered and underwent a four-fold increase from Day 7 to Day 21. Hatching the *A. salina* feedstock in the antibiotic solution significantly reduced its B/H 3.7-fold (Student’s *t*-test, *P*=0.029) (Fig. 1b). One treated Day 21 *E. diaphana* ddPCR sample (gt215) amplified poorly and was excluded from the B/H analysis. ddPCR counts for *A. salina* were also low, but these samples were retained as bacterial load in *A. salina* was not our primary focus.

### Metabarcoding data attributes

Sequencing produced 3747373 raw reads across the 72 *E. diaphana* and six *A. salina* samples (minimum 3825; mean 48043, maximum 88457 reads per sample). After merging, denoising and filtering, 2643644 reads remained (minimum 2404, mean 33891, maximum 55113 reads per sample) and 4628 ASVs were identified. Rarefaction curves for the *E. diaphana* samples plateaued, suggesting that sequencing had captured bacterial diversity (Fig. S2). Seven ASVs, which constituted 0.079 and 0.005% of the bacteria in the *E. diaphana* and *A. salina* respectively, were deemed contaminants by decontam and were removed from the analysis (Table S3). One *E. diaphana* sample (gt215) was removed because it could not be converted to absolute abundance as its B/H ratio could not be determined (see above). Two outlier *E. diaphana* samples were revealed in the nMDS (Fig. S3a) and PCA (Fig. S3b) ordinations and were also removed.

### Changes in bacterial community composition

**Fig. 2.**
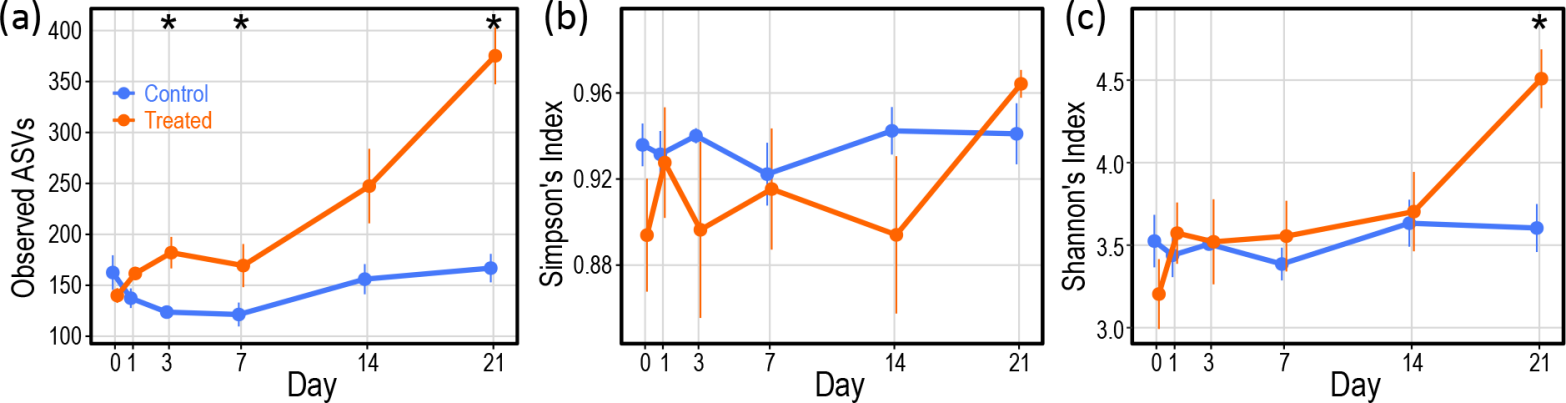
Temporal change in alpha diversity in *E. diaphana.* (**a**) Number of observed ASVs; (**b**) Simpson’s index values; (**c**) Shannon’s index values. For each data point, *n*=5–6. Error bars±1 sem. Asterisks indicate significant differences, α=0.05.

A significant change in the number of observed ASVs in the *E. diaphana* anemones (Fig. 2a) occurred according to treatment (GLS, *χ* ^2^=62.216, *P*<0.001) and time (GLS, *χ* ^2^=42.561, *P*<0.001). A difference between the control and treated *E. diaphana* emerged at Day 3 following an increase in observed ASVs in the treated *E. diaphana* and decrease in the controls (Mann–Whitney, *P*=0.002). However, the decrease in the controls was temporary and there was no significant difference between the number of ASVs observed in the controls at Day 21 compared to Day 0 (Mann–Whitney, *P*=1.000). In contrast, the number of ASVs observed in the treated *E. diaphana* at Day 21 was significantly higher than at Day 0 (Mann–Whitney, *P*=0.008).

A significant temporal change in evenness of the *E. diaphana* bacterial communities, measured according to Simpson’s evenness (Fig. 2b), was detected (GLS, *χ* ^2^=5.934, *P*=0.015). However, this was not supported by post-hoc tests for each timepoint, or in Day 0 versus Day 21 comparisons for each sample type. At the end of the treatment period, overall alpha diversity, measured according to Shannon’s index (Fig. 2c), was significantly higher in the bacterial communities of the treated *E. diaphana* anemones compared to the control *E. diaphana* anemones (Mann–Whitney, *P*=0.004).

**Fig. 3.**
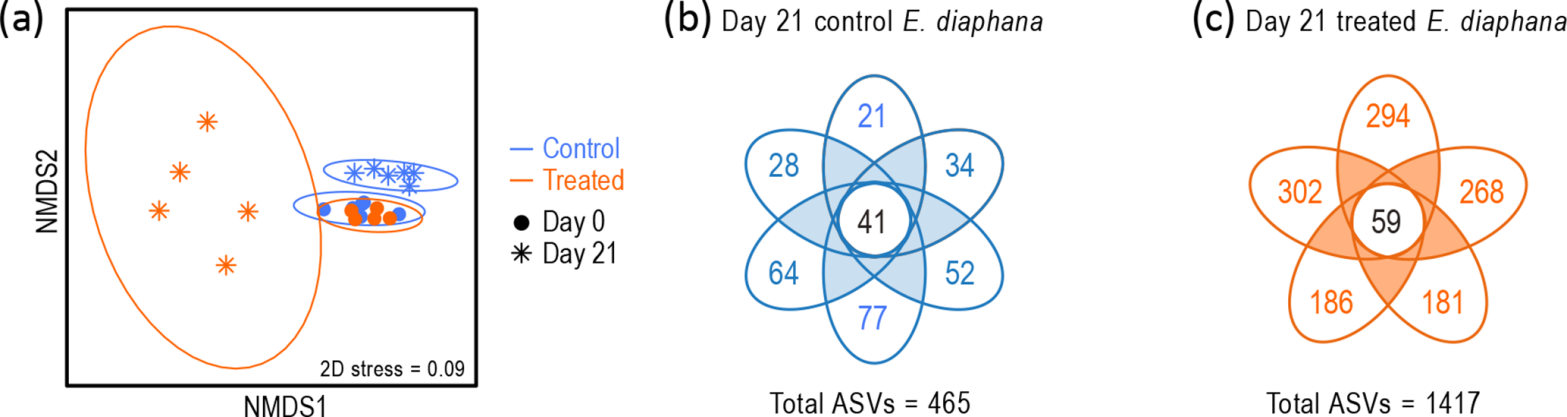
(**a**) nMDS ordination (Bray–Curtis dissimilarity) of bacterial communities in control and treated *E. diaphana* at Day 0 and Day 21 (*n*=5–6; see Fig. S4b for PCA ordination of the Day 0 and Day 21 data, and Fig. S5 for nMDS and PCA ordinations showing all timepoints). (**b, c**) Petal diagrams showing the number of common and unique ASVs in control (*n*=6) and treated (*n*=5) *E. diaphana* at Day 21.

Grouping of Day 0 datapoints in nMDS (Fig. 3a) and PCA (Fig. S4b) ordinations of the *E. diaphana* bacterial community data suggested that the *E. diaphana* anemones were highly similar at the beginning of the experiment, and statistical analyses confirmed they were not significantly different (manyGLM, LRT=492, *P*=0.187). However, by Day 21, the bacterial communities of the control and treated *E. diaphana* anemones had become significantly different from their Day 0 counterparts (control: manyGLM, LRT=1126, *P*=0.003; treated: manyGLM, LRT=1 428, *P*=0.006) and each other (manyGLM, LRT=1725, *P*=0.001). Tight clustering of the Day 21 control *E. diaphana* datapoints suggested that despite undergoing compositional shifts, the bacterial communities of the control *E. diaphana* were still highly uniform after 21 days. In contrast, separation of the datapoints for the Day 21 treated *E. diaphana* indicated decreased uniformity. This may be partially explained by the increase in observed ASVs for the treated *E. diaphana* noted above. A survey of common and unique ASVs in the Day 21 control and treated *E. diaphana* explained this further by showing that, compared to the controls (Fig. 3b), each treated *E. diaphana* (Fig. 3c) harboured a high number of unique ASVs. Together, these data indicated an increase in bacterial beta diversity among the treated *E. diaphana*.

### Antibiotic-tolerant bacteria

Sixteen antibiotic-tolerant ASVs maintained absolute abundances of ≥1000 16S/H × 10^3^ in the treated *E. diaphana* across all timepoints (Table 4; Fig. S6). Despite avoiding elimination, all tolerant ASVs declined in abundance from Day 0 to Day 21, with most declines being significant. Six of the tolerant ASVs were associated with the antibiotic-treated *A. salina* feedstock, and seven were associated with Symbiodiniaceae cells that were isolated from anemones in the UoM *E. diaphana* culture collection and washed to remove extracellular bacteria. All tolerant ASVs were associated with the control *E. diaphana*, indicating their ubiquity among the test anemones. Three tolerant ASVs were members of the genus *Vibrio *.

**Table 4.** Summary of the antibiotic-tolerant ASVs (see Table S4 for full data) Symbiodiniaceae associations are based on data from Maire *et al.* [69], with ‘Close’ and ‘Loose’ associates defined as ‘bacteria tightly attached to the algal cell’s exterior’ and ‘planktonic bacteria’ from the Symbiodiniaceae culture medium, respectively. *P*-values for the Day 0 vs. Day 21 comparisons were calculated using Mann–Whitney *U* tests (α=0.5).

Taxonomic classification				Absolute abundance (16S/H × 10^3^)						Day 0 vs Day 21 *P*-value	Present in *A. salinas*?	Symbiodiniaceae association?		
Class	Order	Family	Genus	Day 0	Day 1	Day 3	Day 7	Day 14	Day 21			Intracellular	Close	Loose
* Gammaproteobacteria *	* Vibrionales *	* Vibrionaceae *	* Vibrio *	6928	6472	4745	1590	1417	1524	0.310		yes	yes	
* Gammaproteobacteria *	*–*	*–*	*–*	20634	28507	9458	4674	2676	1061	0.008				
* Alphaproteobacteria *	* Sphingomonadales *	* Sphingomonadaceae *	* Sphingomonas *	4922	3712	5598	1908	3182	1204	0.016	yes			
* Alphaproteobacteria *	* Rhodobacterales *	* Rhodobacteraceae *	* Thalassobius *	94123	56861	25879	11433	7282	6906	0.032				
* Gammaproteobacteria *	*Coxiellales*	* Coxiellaceae *	* Coxiella *	43074	21771	15844	7171	78850	1903	0.008				
* Gammaproteobacteria *	* Alteromonadales *	* Alteromonadaceae *	*–*	15508	8025	11956	3753	4789	8869	0.095		yes	yes	
* Gammaproteobacteria *	* Vibrionales *	* Vibrionaceae *	* Vibrio *	10338	7333	6792	2439	1248	1238	0.032	yes	yes	yes	yes
* Alphaproteobacteria *	* Sphingomonadales *	* Sphingomonadaceae *	* Erythrobacter *	8616	26317	11092	1290	1002	1754	0.421	yes			
* Deltaproteobacteria *	* Oligoflexales *	* Oligoflexaceae *	*–*	397755	67349	58628	10436	15986	3330	0.008				
* Alphaproteobacteria *	* Rickettsiales *	SM2D12	*–*	9099	9491	12574	3197	1861	1794	0.032				
* Gammaproteobacteria *	* Alteromonadales *	* Marinobacteraceae *	* Marinobacter *	5299	1727	4680	1614	2682	3022	0.548		yes	yes	yes
* Alphaproteobacteria *	* Rhodobacterales *	* Rhodobacteraceae *	* Shimia *	135153	5405	6342	2342	4440	2738	0.032	yes	yes	yes	
* Bacteroidia *	* Chitinophagales *	* Saprospiraceae *	*–*	56148	3157	6652	1988	7482	17836	0.222	yes			
* Gammaproteobacteria *	* Xanthomonadales *	* Xanthomonadaceae *	* Stenotrophomonas *	32799	30630	37369	14014	20619	8801	0.016	yes	yes	yes	yes
* Gammaproteobacteria *	* Vibrionales *	* Vibrionaceae *	* Vibrio *	8025	6303	5786	1775	1185	1311	0.421		yes	yes	
* Alphaproteobacteria *	* Rhodospirillales *	* Terasakiellaceae *	*–*	28068	51153	58769	17803	10451	3742	0.032				

## Discussion

Antibiotic treatment has been used previously to remove [27] or deplete [33] bacteria from *E. diaphana*, but with limited assessment of efficacy, and no information on changes in the bacterial communities. The present study sought to address these gaps by measuring changes in bacterial load and absolute abundance in antibiotic-treated *E. diaphana.* To our knowledge, describing bacterial communities in this way is novel in cnidarian research.

### Antibiotic treatment did not completely eliminate bacteria

Although the treated *E. diaphana* and *A. salina* underwent 6- and 3.7-fold reductions in B/H, respectively, bacteria were not completely eliminated. It is possible that the ddPCR assay amplified DNA from dead bacteria as DNA can persist long after cell death [70, 71], particularly if cells are intact [72], causing bacteria to be overestimated. However, DNA in seawater aquaria has been shown to degrade to levels below detection by ddPCR in ≤94 h [73] and frequent water changes were performed in the present study, which would have removed free DNA and dead bacteria. Culture techniques could be used to test whether positive ddPCR signals originated from viable bacteria, but as previously noted, such analyses are not conclusive due to the uncultivability of many bacteria. Alternatively, viability staining [74] or RT-qPCR/ddPCR of bacterial mRNA [75] could be used to determine whether the remaining bacteria were alive.

Biofilm formation was evident in all wells of the culture plates (Fig. S7). The wells were not cleaned to avoid introducing bacteria or stressing the anemones, but the resulting biofilms undoubtedly impeded elimination of bacteria from the culture environment by shielding bacteria from the antibiotics [76].

The cnidarian surface mucus layer (SML) can be considered a host-associated biofilm [77, 78] that harbours generally transient bacterial communities distinct from the host tissue and surrounding seawater [79]. The SML protects the host by providing a physical barrier against pathogenic bacteria and can have selective antibiotic, but also antibiotic-inhibiting, properties [80]. Although our methods did not allow us to explore correlations between the *E. diaphana* SML and tolerant bacteria, it is possible that the SML assisted the survival of some bacteria.

Bacteria in the treated anemones may have also been protected by intracellular encapsulation or multicellular aggregation within *E. diaphana* tissue. For example, Palincsar *et al.* [81] found that *E. diaphana* exposed to high levels of chloramphenicol (125 mg ml^–1^) or streptomycin (25 mg ml^–1^) for 3 weeks reduced bacterial aggregates by only ~90 and ~50% respectively, thus emphasizing the challenge of using antibiotics to eliminate bacteria from *E. diaphana*.

### Antibiotic treatment increased bacterial diversity

If completely eliminating bacteria from *E. diaphana* is not possible, microbiologically standardized gnotobiotic cultures with low bacterial loads and diversity would still be highly valuable [82]. However, the treated anemones underwent significant increases in bacterial alpha and beta diversity despite reductions in B/H. The increase in bacterial richness in the treated anemones points to a large, diverse pool of bacteria within each anemone that were initially below detection. These bacteria were probably held in check by competition from more abundant bacteria but had higher antibiotic tolerance than those they superseded. The large number of unique ASVs detected in each treated anemone also suggests high bacterial variation between each anemone, which could complicate efforts to generate gnotobiotic *E. diaphana* with uniform bacterial communities.

### Some bacteria tolerated antibiotic treatment

Six of the 16 antibiotic-tolerant ASVs identified in the treated *E. diaphana* were *A. salina* associates, thus implicating *A. salina* as the source, particularly as correlations between *E. diaphana* and *A. salina* feedstock microbiomes have been previously observed [83]. Seven tolerant ASVs were also associated with Symbiodiniaceae cells isolated from *E. diaphana* and washed to remove extracellular ASVs [69]. Therefore, these ASVs may have been located intracellularly within Symbiodiniaceae, which also reside within *E. diaphana* tissue. As this dual encapsulation probably aided the survival of the ASVs by reducing antibiotic exposure, bacterial depletion could be improved by using aposymbiotic anemones (i.e. free of algal symbionts), particularly as the number of Symbiodiniaceae harboured by each anemone (~127 cells mm^–2^ [84]) suggests they could account for a high fraction of bacterial load. However, the ability of *E. diaphana* to withstand both bleaching and antibiotic exposure would need to be tested. The dual encapsulation described above could also explain the survival of a tolerant *Coxiella* ASV, since members of this genus are obligate intracellular parasites [85]. Three tolerant ASVs were members of *Vibrio *, a genus often associated with microbiome dysbiosis and disease in cnidarians [86], that has many members in the marine environment known to possess antibiotic resistance [87]. The presence of bacteria that are not only tolerant but also antibiotic resistant may make generation of gnotobiotic *E. diaphana* by antibiotic treatment alone difficult. Four tolerant ASVs belonged to taxonomic groups (* Thalassobius *, *Saprospiraceae *, *Marinobacter* and *Oligoflexaceae *) with members previously identified as core *E. diaphana* associates [83]. Among these, the family *Saprospiraceae* is noteworthy as it contains species that are frequently found in plastic-associated marine biofilms, highlighting the role probably played by biofilms in the survival of the tolerant ASVs identified in our study [88–90].

### Recommendations for improved bacterial depletion in *E. diaphana*

Based on our findings, we provide the following recommendations for future gnotobiotic *E. diaphana* work. First and foremost, the increase in B/H in the control anemones suggests that even under sterile conditions, maintaining vessel cleanliness is essential to remove biofilms and cellular debris that can harbour and protect bacteria. Indeed, since the present study was conducted, rearing *E. diaphana* under sterile conditions with regular cleaning has been shown to substantially reduce bacterial alpha diversity [91]. Second, due to the correlation between the *E. diaphana* and *A. salina* bacteria, proper feedstock sterilization is vital. This could be achieved by hatching the *A. salina* in higher antibiotic concentrations or by using chemical decapsulation as described by Sorgeloos *et al.* [92] and employed by Costa *et al.* [33]. However, care would be required not to introduce more antibiotics through feeding than could be tolerated by *E. diaphana.* Third, the survival of all treated *E. diaphana* suggests that prolonged antibiotic treatment is viable, and therefore bacterial load and diversity reduction via long-term exposure should be explored. Fourth, the recent discovery that Symbiodiniaceae contain intracellular bacteria suggests the use of aposymbiotic anemones could help reduce bacterial load and we encourage exploration of this approach. Finally, methods that address the increased bacterial beta diversity we observed in antibiotic-treated *E. diaphana* should be investigated. These include generating *E. diaphana* cultures from a single founder anemone, or alternatively from pedal lacerates or cell fragments (i.e. artificial lacerates) since smaller treatment subjects probably harbour fewer bacteria and few, or no, bacterial aggregates. Taken further, sterilization of fertilized eggs or larvae, as performed on other organisms [93–95], could provide the ideal path towards gnotobiotic *E. diaphana*, although closing the cycle of sexual reproduction in lab-reared *E. diaphana* has proven elusive [96]

## Conclusion

Antibiotic exposure for 3 weeks significantly reduced the bacterial load of *E. diaphana*, but also increased the complexity and variability of the anemones’ bacterial communities, and hence they could not be defined as gnotobiotic. Extended treatment could improve bacterial depletion, providing culture vessels and food are sterile. However, the efficacy of antibiotic treatment could ultimately be limited by the diversity of native bacteria, some of which probably possess antibiotic tolerance or resistance. Therefore, using treatment subjects with naïve bacterial communities might be needed to create gnotobiotic *E. diaphana* which, if produced, would represent a substantial advance in cnidarian symbiosis research.

## Supplementary Data

Supplementary material 1
