# Supplementary material for: Antibiotics reduce bacterial load in Exaiptasia diaphana, but biofilms hinder its development as a gnotobiotic coral model
Source: Access Microbiol. 2022 Jan 24;4(1):000314. doi: 10.1099/acmi.0.000314 (PMC8895603; doi:10.1099/acmi.0.000314)
Supplement: Supplementary material 1 [file acmi-4-0314-s001.pdf]

Table S1: ddPCR data for bacterial (16S) reactions. NTC = no template control.

Sample	Condition	Day	Sample Type	Concentration	Copies/Well	Positives	Negatives	Accepted Droplets
NTC	–	–	–	6.2	124	75	14197	14272
gc01	control	0	anemone	44.8	896	549	14146	14695
gc02	control	0	anemone	34.7	694	358	11964	12322
gc03	control	0	anemone	98.0	1960	1092	12575	13667
gc04	control	0	anemone	17.6	352	197	13074	13271
gc05	control	0	anemone	213.0	4260	2338	11783	14121
gc06	control	0	anemone	662.0	13240	5928	7845	13773
gc11	control	1	anemone	104.0	2080	1254	13520	14774
gc12	control	1	anemone	42.7	854	506	13696	14202
gc13	control	1	anemone	51.2	1024	607	13658	14265
gc14	control	1	anemone	160.0	3200	1723	11827	13550
gc15	control	1	anemone	62.0	1240	804	14864	15668
gc16	control	1	anemone	59.2	1184	723	13999	14722
gc31	control	3	anemone	238.0	4760	2609	11647	14256
gc32	control	3	anemone	499.0	9980	5057	9583	14640
gc33	control	3	anemone	80.0	1600	944	13364	14308
gc34	control	3	anemone	88.0	1760	1049	13522	14571
gc35	control	3	anemone	144.0	2880	1706	13122	14828
gc36	control	3	anemone	88.0	1760	1141	14734	15875
gc71	control	7	anemone	340.0	6800	3548	10597	14145
gc72	control	7	anemone	294.0	5880	3015	10628	13643
gc73	control	7	anemone	157.0	3140	1777	12455	14232
gc74	control	7	anemone	81.3	1626	1067	14918	15985
gc75	control	7	anemone	49.7	994	624	14458	15082
gc76	control	7	anemone	90.0	1800	1082	13536	14618
gc141	control	14	anemone	176.0	3520	1912	11856	13768
gc142	control	14	anemone	512.0	10240	4993	9152	14145
gc143	control	14	anemone	518.0	10360	4535	8198	12733
gc144	control	14	anemone	1632.0	32640	10688	3560	14248
gc145	control	14	anemone	93.0	1860	1087	13241	14328
gc146	control	14	anemone	110.0	2200	1306	13277	14583
gc211	control	21	anemone	505.0	10100	4700	8766	13466
gc212	control	21	anemone	1494.0	29880	10720	4188	14908
gc213	control	21	anemone	1008.0	20160	7987	5895	13882
gc214	control	21	anemone	287.0	5740	3153	11407	14560
gc215	control	21	anemone	771.0	15420	6969	7534	14503
gc216	control	21	anemone	175.0	3500	1923	12023	13946
gt01	treated	0	anemone	139.0	2780	1734	13837	15571
gt02	treated	0	anemone	33.8	676	388	13314	13702

gt03	treated	0	anemone	303.0	6060	3052	10379	13431
gt04	treated	0	anemone	28.0	560	355	14762	15117
gt05	treated	0	anemone	140.0	2800	1655	13058	14713
gt06	treated	0	anemone	26.3	526	342	15103	15445
gt11	treated	1	anemone	12.3	246	168	16003	16171
gt12	treated	1	anemone	22.2	444	286	15045	15331
gt13	treated	1	anemone	112.0	2240	1495	14999	16494
gt14	treated	1	anemone	34.8	696	448	14936	15384
gt15	treated	1	anemone	72.8	1456	954	14948	15902
gt16	treated	1	anemone	29.4	588	350	13833	14183
gt31	treated	3	anemone	59.7	1194	730	14023	14753
gt32	treated	3	anemone	67.6	1352	853	14424	15277
gt33	treated	3	anemone	87.5	1743	767	10741	10741
gt34	treated	3	anemone	34.3	686	444	14992	15436
gt35	treated	3	anemone	36.7	734	472	14898	15370
gt36	treated	3	anemone	52.0	1040	654	14479	15133
gt71	treated	7	anemone	16.8	336	195	13556	13751
gt72	treated	7	anemone	25.6	512	328	14938	15266
gt73	treated	7	anemone	9.2	184	102	13003	13105
gt74	treated	7	anemone	16.4	328	212	15070	15282
gt75	treated	7	anemone	37.5	750	444	13708	14152
gt76	treated	7	anemone	17.6	352	198	13106	13304
gt141	treated	14	anemone	16.7	334	221	15432	15653
gt142	treated	14	anemone	31.5	630	365	13442	13807
gt143	treated	14	anemone	22l5	450	286	14840	15126
gt144	treated	14	anemone	28.5	570	280	11400	11680
gt145	treated	14	anemone	20.2	404	236	13640	13876
gt146	treated	14	anemone	18.4	368	237	15023	15260
gt211	treated	21	anemone	11.5	230	141	14333	14474
gt212	treated	21	anemone	24.3	486	347	16597	16944
gt213	treated	21	anemone	52.4	1048	675	14814	15489
gt214	treated	21	anemone	11.7	234	131	13075	13206
gt215	treated	21	anemone	4.4	88	55	14582	14637
gt216	treated	21	anemone	14.3	286	190	15501	15691
gca1	control	–	artemia	8.3	166	133	15916	16029
gca2	control	–	artemia	6.7	134	85	14913	14998
gca3	control	–	artemia	5.6	112	64	13432	13496
gta1	treated	–	artemia	4.9	98	58	13909	13967
gta2	treated	–	artemia	4.5	90	55	14351	14406
gta3	treated	–	artemia	3.0	60	22	8498	8520

Table S2: ddPCR data for host (*Ef1-α* or *ß-actin*) reactions. NTC = no template control.

Sample	Condition	Day	Sample Type	Concentration	Copies/Well	Positives	Negatives	Accepted Droplets
NTC	–	–	–	No Call	0	0	15301	15301
gc01	control	0	anemone	1275	25500	9198	4702	13900
gc02	control	0	anemone	1246	24920	10187	5405	15592
gc03	control	0	anemone	1734	34680	10684	3174	13858
gc04	control	0	anemone	213	4260	2142	10779	12921
gc05	control	0	anemone	1802	36040	11070	3053	14123
gc06	control	0	anemone	1874	37480	11814	3015	14829
gc11	control	1	anemone	1640	32800	10874	3588	14462
gc12	control	1	anemone	1707	34140	10020	3067	13087
gc13	control	1	anemone	1158	23160	9635	5746	15381
gc14	control	1	anemone	1566	31320	10402	3735	14137
gc15	control	1	anemone	1022	20440	8331	6023	14354
gc16	control	1	anemone	1392	27840	9419	4160	13579
gc31	control	3	anemone	2205	44100	12177	2208	14385
gc32	control	3	anemone	1494	29880	10217	3990	14207
gc33	control	3	anemone	2940	58800	13088	1175	14263
gc34	control	3	anemone	2870	57400	12874	1233	14107
gc35	control	3	anemone	1675	33500	10993	3487	14480
gc36	control	3	anemone	1892	37840	11193	2804	13997
gc71	control	7	anemone	1293	25860	8618	4305	12923
gc72	control	7	anemone	2092	41840	12258	2491	14749
gc73	control	7	anemone	1549	30980	10555	3866	14421
gc74	control	7	anemone	1261	25220	8869	4616	13485
gc75	control	7	anemone	1924	38480	12306	2979	15285
gc76	control	7	anemone	2540	50800	12420	1628	14048
gc141	control	14	anemone	1640	32800	10313	3401	13714
gc142	control	14	anemone	945	18900	7437	6030	13467
gc143	control	14	anemone	1805	36100	10229	2813	13042
gc144	control	14	anemone	2730	54600	11338	1231	12569
gc145	control	14	anemone	2172	43440	12293	2304	14597
gc146	control	14	anemone	1556	31120	10149	3686	13835
gc211	control	21	anemone	1632	32640	9766	3251	13071
gc212	control	21	anemone	1686	33720	10474	3283	13757
gc213	control	21	anemone	1491	29820	9425	3696	13121
gc214	control	21	anemone	3030	60600	13406	1108	14514
gc215	control	21	anemone	1383	27660	9471	4227	13698
gc216	control	21	anemone	1872	37440	10984	2809	13793
gt01	treated	0	anemone	1536	30720	10638	3954	14592
gt02	treated	0	anemone	952	19040	7571	6072	13643

gt03	treated	0	anemone	1543	30860	10915	4024	14939
gt04	treated	0	anemone	1120	22400	8954	5625	14579
gt05	treated	0	anemone	868	17360	7323	6709	14032
gt06	treated	0	anemone	1192	23840	10528	6003	16531
gt11	treated	1	anemone	277	5540	3509	13200	16709
gt12	treated	1	anemone	1949	38980	11327	2669	13996
gt13	treated	1	anemone	1884	37680	12694	3205	15899
gt14	treated	1	anemone	1415	28300	10027	4306	14333
gt15	treated	1	anemone	1471	29420	11946	4793	16739
gt16	treated	1	anemone	1372	27440	9770	4423	14193
gt31	treated	3	anemone	1237	24740	9339	5019	14358
gt32	treated	3	anemone	4180	83600	14192	419	14611
gt33	treated	3	anemone	1690	33800	10835	3379	14214
gt34	treated	3	anemone	1967	39340	11679	2702	14381
gt35	treated	3	anemone	2181	43620	12464	2315	14779
gt36	treated	3	anemone	1659	33180	11222	3626	14848
gt71	treated	7	anemone	1551	31020	10465	3825	14290
gt72	treated	7	anemone	1484	29680	10988	4341	15329
gt73	treated	7	anemone	1275	25500	9010	4607	13617
gt74	treated	7	anemone	1367	27340	9945	4530	14475
gt75	treated	7	anemone	2049	40980	11449	2432	13881
gt76	treated	7	anemone	1805	36100	11161	3069	14230
gt141	treated	14	anemone	1310	26200	9333	4561	13894
gt142	treated	14	anemone	1308	26160	9622	4715	14337
gt143	treated	14	anemone	2580	51600	13600	1702	15302
gt144	treated	14	anemone	1811	36220	12124	3310	15434
gt145	treated	14	anemone	1779	35580	11440	3235	14675
gt146	treated	14	anemone	1333	26660	9536	4532	14068
gt211	treated	21	anemone	1045	20900	8216	5740	13956
gt212	treated	21	anemone	1795	35900	11126	3092	14218
gt213	treated	21	anemone	1036	20720	8776	6213	14989
gt214	treated	21	anemone	1309	26180	8027	3931	11958
gt215	treated	21	anemone	142	2840	1707	13270	14977
gt216	treated	21	anemone	1534	30680	10787	4021	14808
gca1	control	–	artemia	62	1244	848	15612	16460
gca2	control	–	artemia	84	1680	1005	13560	14565
gca3	control	–	artemia	83	1660	316	11201	12017
gta1	treated	–	artemia	167	3340	1902	12439	14341
gta2	treated	–	artemia	165	3300	1915	12735	14650
gta3	treated	–	artemia	155	3100	1588	11242	12830

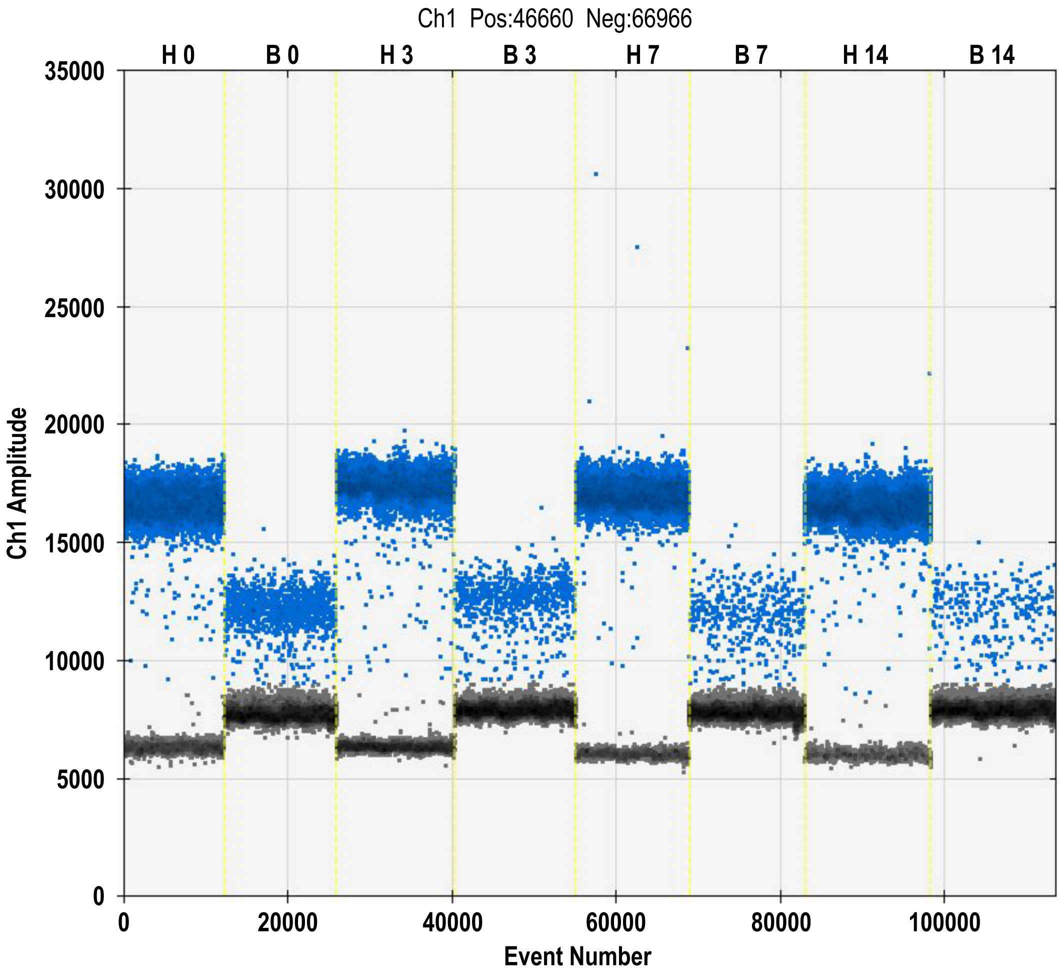

Fig. S1: ddPCR fluorescence output for Bacteria-Host reaction pairs from four representative treated anemones at (left-to-right) days 0, 3 ,7 and 14. Blue datapoints indicate positive reactions; black datapoints indicate negative reactions. A decrease in the positive ddPCR signals for the bacterial samples (B0, B3, B7, B14) illustrates the reduction in bacteria in the treated anemones. The bacterial ddPCR samples generated a moderate amount of rain, however QuantaSoft automatically separated positive and negative signals in all 158 samples except one, for which a manual threshold was set.

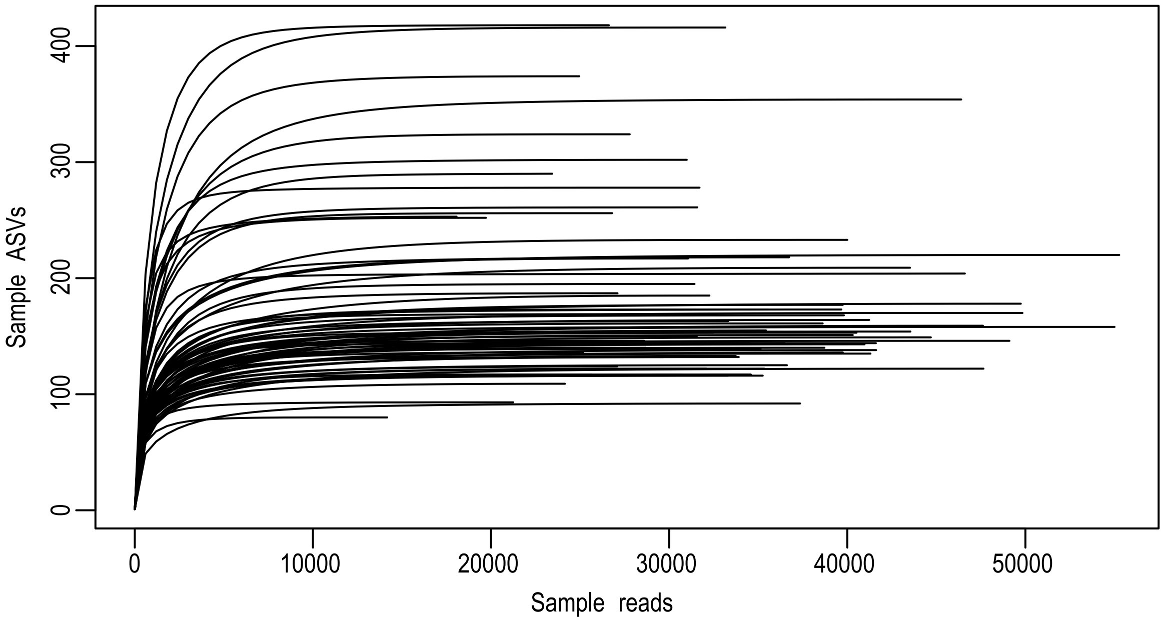

Fig. S2: Rarefaction curves for the raw *E. diaphana* 16S rRNA gene metabarcoding data. All curves plateaued indicating that sequencing captured bacterial community diversity in each sample. For analyses, the data were rarefied to 48 176 ASV counts per sample after conversion to absolute abundance using the B/H ratios.

Table S3: Putative contaminant ASVs removed from the dataset. Seven ASVs potentially introduced during sample processing were identified with the R package decontam using data from the negative control samples. All were removed from the dataset.

	Phylum	Class	Order	Family	Genus	*E.diaphana* Rel. ab. (%)	*A.salina* Rel. ab. (%)
1	Proteobacteria	Gammaproteobacteria				0.0025	0.0000
2	Proteobacteria	Alphaproteobacteria	Rhizobiales	Rhizobiaceae	*Mesorhizobium*	0.0007	0.0000
3	Proteobacteria	Alphaproteobacteria	Rhizobiales	Xanthobacteraceae	*Bradyrhizobium*	0.0041	0.0000
4	Proteobacteria	Alphaproteobacteria	Rhizobiales	Xanthobacteraceae	*Afipia*	0.0081	0.0051
5	Bacteroidetes	Bacteroidia	Sphingobacteriales	Sphingobacteriaceae	*Sphingobacterium*	0.0003	0.0000
6	Proteobacteria	Gammaproteobacteria	Betaproteobacteriales	Burkholderiaceae	*Ralstonia*	0.0601	0.0000
7	Proteobacteria	Gammaproteobacteria	Betaproteobacteriales	Burkholderiaceae		0.0033	0.0000
						0.0791	0.0051

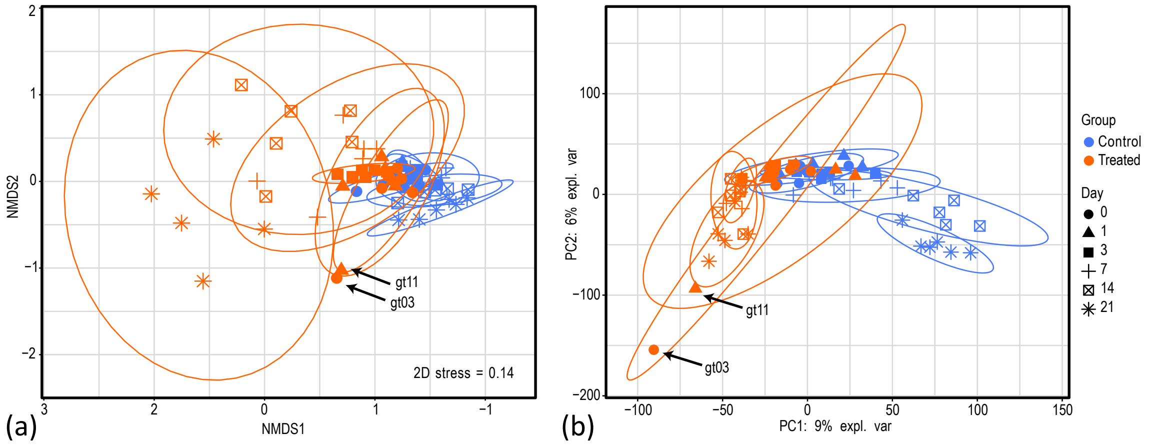

Fig. S3: (a) nMDS ordination (Bray-Curtis dissimilarity), and (b) PCA ordination (CLR-transformed data) of the *E. diaphana* bacterial communities. Two outlier samples (indicated) were removed from the dataset. The nMDS x-axis has been reversed to aid comparison.

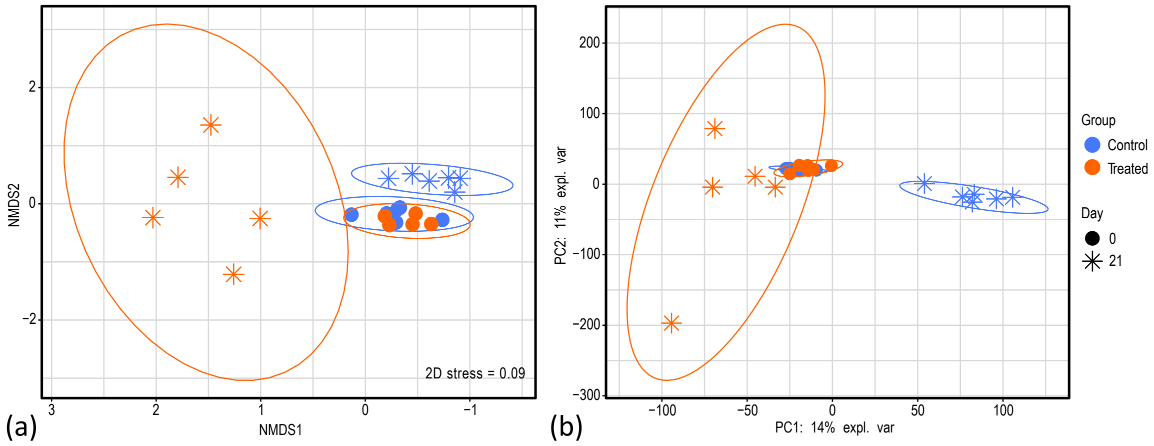

Fig. S4: (a) nMDS ordination (Bray-Curtis dissimilarity), and (b) PCA ordination (CLR-transformed data) of the *E. diaphana* bacterial communities at Day 0 and Day 21.

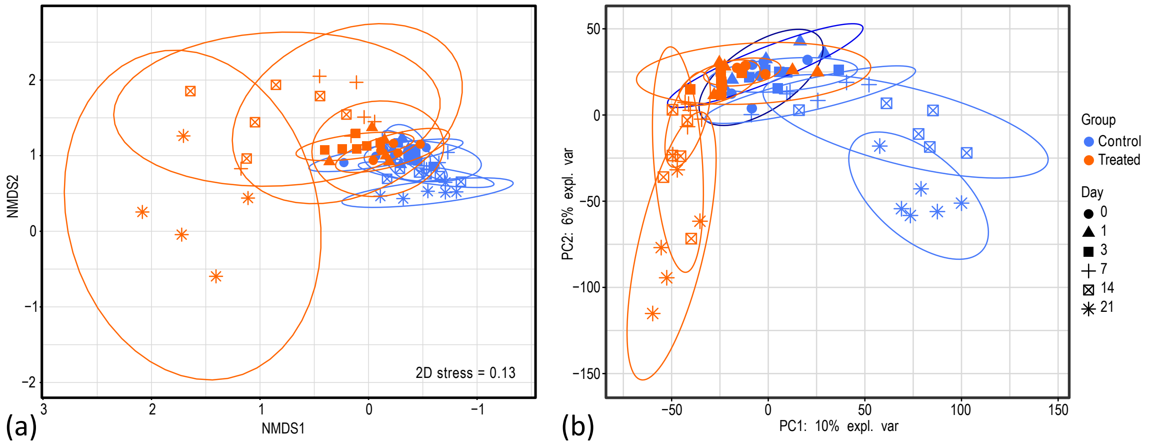

Fig. S5: (a) nMDS ordination (Bray-Curtis dissimilarity), and (b) PCA ordination (CLR-transformed data) of the *E. diaphana* bacterial communities across all timepoints.

Table S4: Summary of the antibiotic tolerant ASVs. *p*-values for the Day 0 vs Day 21 comparisons were calculated using Mann-Whitney U tests. Significant *p*-values are shown in bold (α = 0.5).

ASV	Absolute abundance (16S $H^{-1} \times 10^3$)												Day 0 vs Day 21	Present in	Symbiodiniaceae association? *		
	Day 0	Day 1	Day 3	Day 7	Day 14	Day 21	Phylum	Class	Order	Family	Genus	Species	*p* -value	*A. Salinas* ?	Intracellular	Close	Loose
613a6844484c3b2f90d38399d2698624	6928	6472	4745	1590	1417	1524	Proteobacteria	Gammaproteobacteria	Vibrionales	Vibrionaceae	*Vibrio*	–	0.310		yes	yes	
2832a0bcf6ae6d1d6c9e67822301c207	20634	28507	9458	4674	2676	1061	Proteobacteria	Gammaproteobacteria	–	–	–	–	**0.008**				
dc984a63d7d685f878587c30e0d8f18f	4922	3712	5598	1908	3182	1204	Proteobacteria	Alphaproteobacteria	Sphingomonadales	Sphingomonadaceae	*Sphingomonas*	–	**0.016**	yes			
0142e3efbe4c804a7b44f9ddbfc319a6	94123	56861	25879	11433	7282	6906	Proteobacteria	Alphaproteobacteria	Rhodobacterales	Rhodobacteraceae	*Thalassobius*	–	**0.032**				
4cf05c2c773187a18f17016856d1d3c6	43074	21771	15844	7171	78850	1903	Proteobacteria	Gammaproteobacteria	Coxiellales	Coxiellaceae	*Coxiella*	–	**0.008**				
e3c9fb8d8e882e9f6d98ff1d7c32f03f	15508	8025	11956	3753	4789	8869	Proteobacteria	Gammaproteobacteria	Alteromonadales	Alteromonadaceae	–	–	0.095		yes	yes	
ae8a6381f4e78d50e3fad0e2faad8ea0	10338	7333	6792	2439	1248	1238	Proteobacteria	Gammaproteobacteria	Vibrionales	Vibrionaceae	*Vibrio*	–	**0.032**	yes	yes	yes	yes
11bb58be038678b5e2b1878c01140c05	8616	26317	11092	1290	1002	1754	Proteobacteria	Alphaproteobacteria	Sphingomonadales	Sphingomonadaceae	*Erythrobacter*	–	0.421	yes			
682e5342211c704576db92e0bb567c8e	397755	67349	58628	10436	15986	3330	Proteobacteria	Deltaproteobacteria	Oligoflexales	Oligoflexaceae	–	–	**0.008**				
3cf1c3677b74a3ab095f77b115f0ec14	9099	9491	12574	3197	1861	1794	Proteobacteria	Alphaproteobacteria	Rickettsiales	SM2D12	–	–	**0.032**				
e0cc6a95596c1068ec99eb67cea8d93e	5299	1727	4680	1614	2682	3022	Proteobacteria	Gammaproteobacteria	Alteromonadales	Marinobacteraceae	*Marinobacter*	–	0.548		yes	yes	yes
53818a706e38c1584f139d2f90fbd8df	135153	5405	6342	2342	4440	2738	Proteobacteria	Alphaproteobacteria	Rhodobacterales	Rhodobacteraceae	*Shimia*	–	**0.032**	yes	yes	yes	
107e04ddc4519998efd0c7f0d4a3c619	56148	3157	6652	1988	7482	17836	Bacteroidetes	Bacteroidia	Chitinophagales	Saprospiraceae	–	–	0.222	yes			
acd191a5f307d0579b3126166f102435	32799	30630	37369	14014	20619	8801	Proteobacteria	Gammaproteobacteria	Xanthomonadales	Xanthomonadaceae	*Stenotrophomonas*	–	**0.016**	yes	yes	yes	yes
26a930ff8d7ae487ae9b3beaf07b53ca	8025	6303	5786	1775	1185	1311	Proteobacteria	Gammaproteobacteria	Vibrionales	Vibrionaceae	*Vibrio*	–	0.421		yes	yes	
05ee672c13ca2e0e6c44a8c8ec463c05	28068	51153	58769	17803	10451	3742	Proteobacteria	Alphaproteobacteria	Rhodospirillales	Terasakiellaceae	–	–	**0.032**				

* Based on data from Maire (2021)

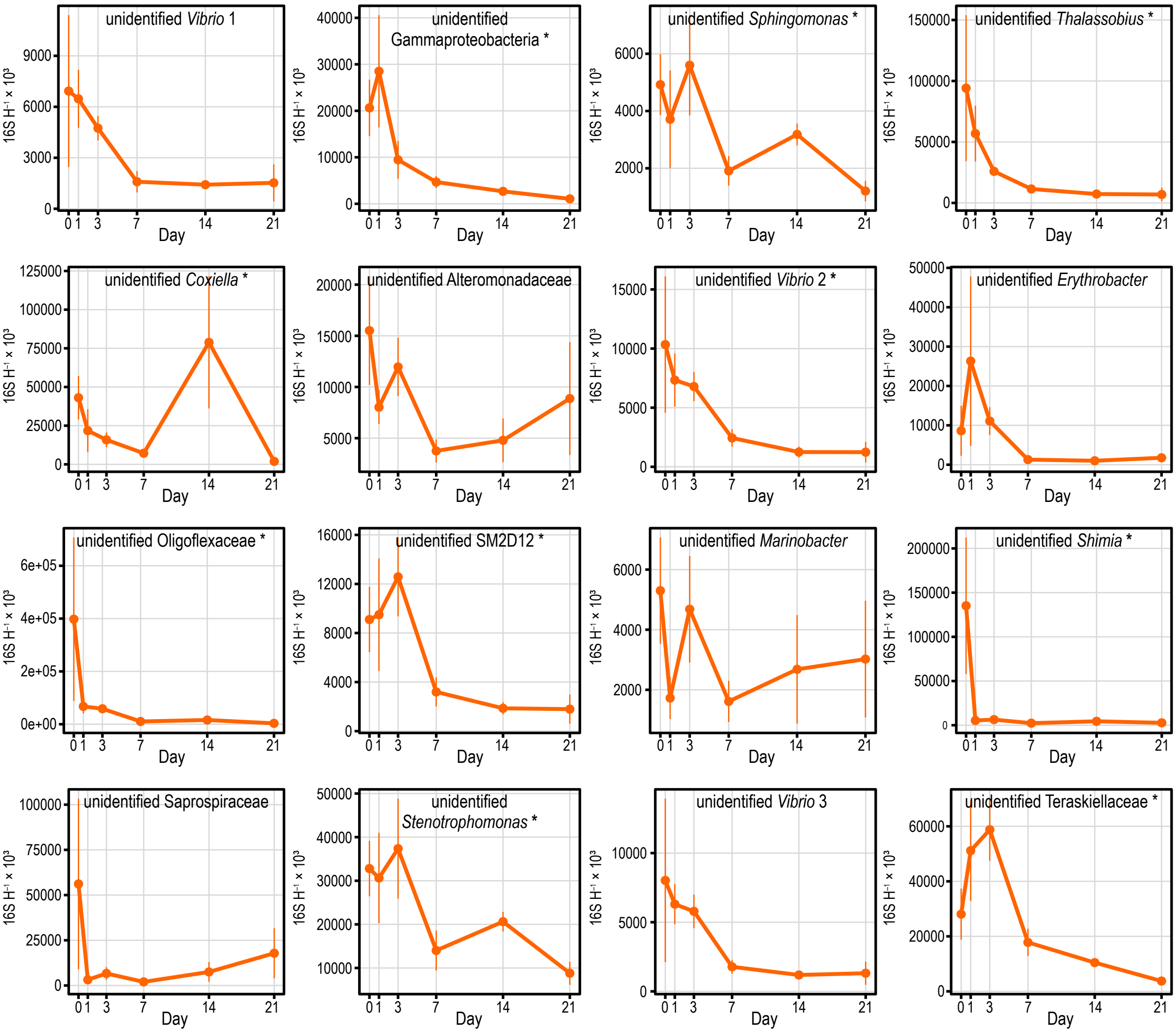

Fig. S6: Antibiotic-tolerant bacterial ASVs with absolute abundances ≥1000 16S $H^{-1} \times 10^3$ at all timepoints in the antibiotic-treated *E. diaphana*. For each datapoint, n = 5-6. Error bars ± 1 SEM. Asterisks indicate tolerant ASVs with significantly different abundances at Day 21 compared to Day 0 (α = 0.5).

(a) (b)

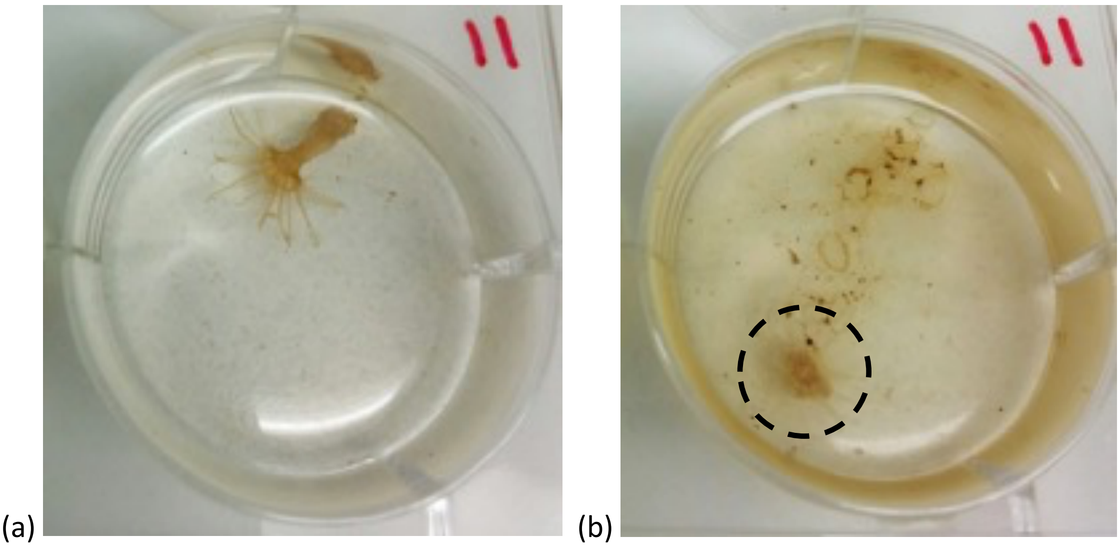

Fig. S7: (a) *E. diaphana* anemone at Day 0, before antibiotic treatment; (b) Anemone (circled) after antibiotic treatment for Day 21 days. Biofilms on the sides of the well and cellular debris (deposited as the anemone traversed the well) are visible. The amount of cellular debris in each well varied depending on the mobility of the anemones, but biofilms accumulated in all wells.

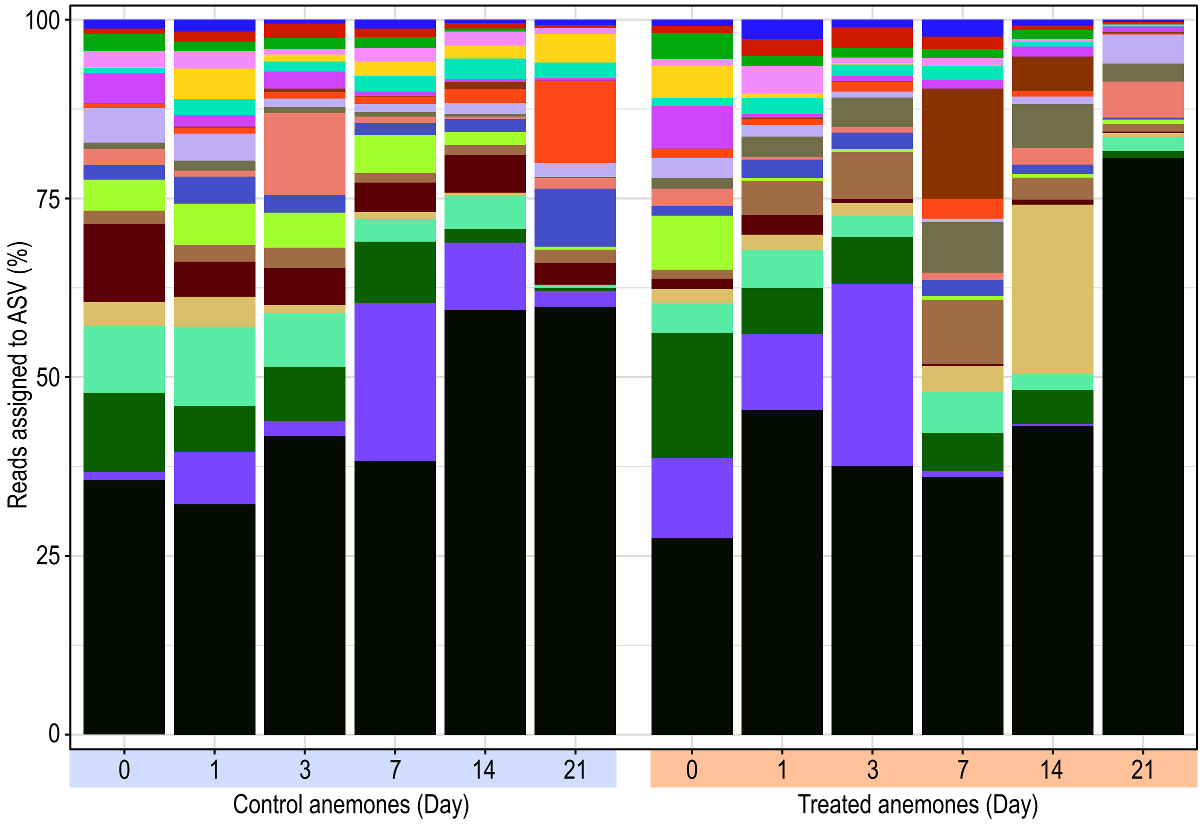

	ASV	Phylum	Class	Order	Family	Genus	Species	Tolerant ASV?
	853d62f828d0f67cdd22a2f0c9e145a5	Proteobacteria	Deltaproteobacteria	Oligoflexales	Oligoflexaceae	–	–	
	77972cd35884d5c610062f44d668e9fb	Proteobacteria	Gammaproteobacteria	Francisellales	Francisellaceae	*Francisella*	–	
	2e371beb95bfbb6eb92ccd5987360ca4	Proteobacteria	Alphaproteobacteria	Rhizobiales	Stappiaceae	*Labrenzia*	–	
	53818a706e38c1584f139d2f90fbd8df	Proteobacteria	Alphaproteobacteria	Rhodobacterales	Rhodobacteraceae	–	–	yes
	9eee17f08d33b67a62e3ee0d5e633918	Proteobacteria	Deltaproteobacteria	PB19	–	–	–	
	2832a0bcf6ae6d1d6c9e67822301c207	Proteobacteria	Gammaproteobacteria	–	–	–	–	yes
	5eea17e793b081eefa9ce6e86a408317	Proteobacteria	Alphaproteobacteria	Rhizobiales	Rhizobiaceae	–	–	
	dd5a59afc2cff7f8e78604872e07bcab	Proteobacteria	Alphaproteobacteria	Rhizobiales	Stappiaceae	*Labrenzia*	–	
	107e04ddc4519998efd0c7f0d4a3c619	Bacteroidetes	Bacteroidia	Chitinophagales	Saprospiraceae	–	–	yes
	0fc85aac1e7f4a6de518d7dd44b72e9d	Proteobacteria	Alphaproteobacteria	Rhodospirillales	Terasakiellaceae	–	–	
	8a45e0b7d52715cbb937eaa0aaebd098	Proteobacteria	Alphaproteobacteria	Rhodobacterales	Rhodobacteraceae	–	–	
	062b090c083845612e0ed7dc400c9106	Proteobacteria	Deltaproteobacteria	Myxococcales	Nannocystaceae	–	–	
	733bb7586fe47d0dc7519a3ab4e1d6e8	Proteobacteria	Alphaproteobacteria	Rhizobiales	Stappiaceae	*Labrenzia*	–	
	acd191a5f307d0579b3126166f102435	Proteobacteria	Gammaproteobacteria	Xanthomonadales	Xanthomonadaceae	*Stenotrophomonas*	–	yes
	05ee672c13ca2e0e6c44a8c8ec463c05	Proteobacteria	Alphaproteobacteria	Rhodospirillales	Terasakiellaceae	–	–	yes
	4cf05c2c773187a18f17016856d1d3c6	Proteobacteria	Gammaproteobacteria	Coxiellales	Coxiellaceae	*Coxiella*	–	yes
	29e58ca9bdd079d531e2aed3eb1e413a	Spirochaetes	Spirochaetia	Spirochaetales	Spirochaetaceae	*Spirochaeta 2*	–	
	0142e3efbe4c804a7b44f9ddbfc319a6	Proteobacteria	Alphaproteobacteria	Rhodobacterales	Rhodobacteraceae	*Thalassobius*	–	yes
	682e5342211c704576db92e0bb567c8e	Proteobacteria	Deltaproteobacteria	Oligoflexales	Oligoflexaceae	–	–	yes
	da6cc46660ce08372dadadde2ebf11ca	Proteobacteria	Alphaproteobacteria	Rhodobacterales	Rhodobacteraceae	*Ruegeria*	–	yes
	Other							

Fig. S8: Changes in relative abundance for the 20 ASVs with the highest relative abundance across all samples and timepoints. For each bar, *n* = 5-6. ASVs are organised least-to-most abundant, top-to-bottom in the figure key. Nine of the ASVs were also identified as antibiotic-tolerant (see Table S4), with maintenance of those bacteria potentially influenced by their high relative abundances. An increase in the category "Other" illustrated the increase in bacterial diversity for both control and antibiotic-treated *E. diaphana* as their bacterial communities became dominated by more low abundance bacteria.
